# Translating the COVID-19 epidemiological situation into policies and measures: the Belgian experience

**DOI:** 10.3389/fpubh.2024.1306361

**Published:** 2024-04-05

**Authors:** Géraldine De Muylder, Valeska Laisnez, Giulietta Stefani, Caroline Boulouffe, Christel Faes, Naïma Hammami, Pierre Hubin, Geert Molenberghs, Jasper Sans, Cecile van de Konijnenburg, Stefaan Van der Borght, Ruben Brondeel, Jorgen Stassijns, Tinne Lernout

**Affiliations:** ^1^Department of Epidemiology and Public Health, Sciensano, Brussels, Belgium; ^2^Agence pour une Vie de Qualité (AVIQ), Charleroi, Belgium; ^3^Data Science Institute, I-BioStat, Hasselt University, Hasselt, Belgium; ^4^Department of Care, Team Infection Prevention and Vaccination, Brussels, Belgium; ^5^Faculty of Medicine, Department of Public Health and Primary Care, L-BioStat, Leuven, Belgium; ^6^Department of Infectious Disease Prevention, Brussels, Belgium; ^7^Federal Public Service - Public Health - Risk Management Group, Brussels, Belgium; ^8^Crisis Management and Strategy, Sciensano, Brussels, Belgium

**Keywords:** data, policies, barometer, risk management, COVID-19, management tool

## Abstract

The COVID-19 pandemic led to sustained surveillance efforts, which made unprecedented volumes and types of data available. In Belgium, these data were used to conduct a targeted and regular assessment of the epidemiological situation. In addition, management tools were developed, incorporating key indicators and thresholds, to define risk levels and offer guidance to policy makers. Categorizing risk into various levels provided a stable framework to monitor the COVID-19 epidemiological situation and allowed for clear communication to authorities. Although translating risk levels into specific public health measures has remained challenging, this experience was foundational for future evaluation of the situation for respiratory infections in general, which, in Belgium, is now based on a management tool combining different data sources.

## Introduction

Following the WHO International Health Regulations from 2005 “to prevent, protect against, control and provide a public health response to the international spread of disease in ways that are commensurate with and restricted to public health risks,” Belgian authorities established the Risk Assessment Group (RAG) and the Risk Management group (RMG) in 2007 (1, 2). The importance of these structures was confirmed in 2013 by the Decision No 1082/2013/EU of the European Parliament and European Council on serious cross-border threats to health.

Identification of potential threats to public health in Belgium is performed by the Belgian Health Institute, Sciensano, and is based on epidemic intelligence and systematic decoding of signals identified through epidemiological surveillance. These public health threats can be of microbiological, chemical or environmental origin. The RAG has the responsibility, upon identification of a possible threat to public health, to (i) evaluate the threat, (ii) assess the risk posed to public health for the Belgian population, (iii) propose measures to limit or control the threat (within the public health domain) and (iv) follow-up risks and interventions. The RAG is coordinated by Sciensano and is composed of representatives of the health authorities (infection prevention and control departments), the Belgian Superior Health Council and professionals invited based on their expertise (epidemiologists, clinicians, microbiologists, hygienists, environmental specialists, biostatisticians, etc).

**Figure 1:**
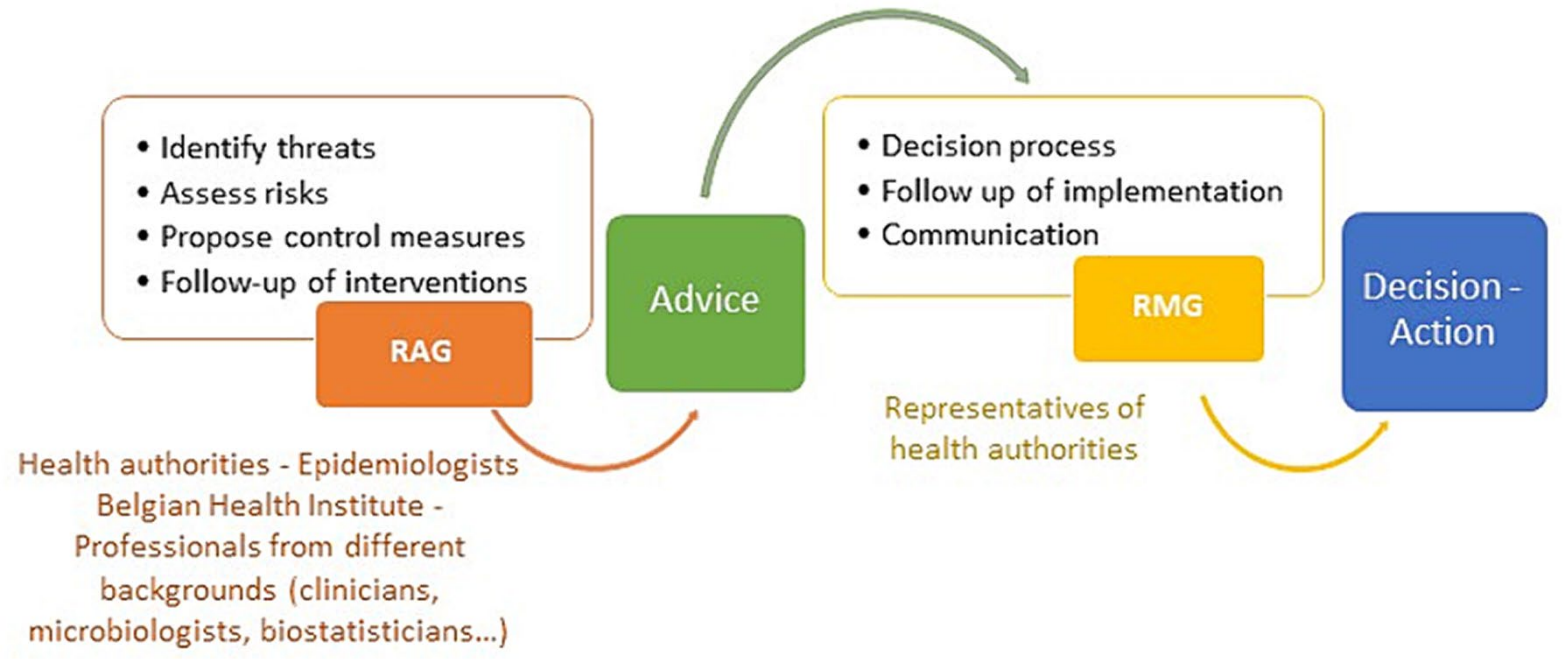
Description of the risk assessment and decision making process in Belgium. Note that during the COVID-19 crisis additional bodies were involved such as the GEMS or the Commissariat.

Recommendations proposed by the RAG are presented to the RMG, which is composed of representatives of health authorities (administration and ministries), and which is in charge of taking decisions on measures to limit the impact or control the threat, implement these measures and communicate them (Figure 1).

During the COVID-19 pandemic, a substantial number of advice requests were issued to the RAG on a wide range of topics, including testing strategy and measures for cases and contacts (3). In addition, since August 2020, the RAG has made a weekly evaluation of the COVID-19 epidemiological situation, based on data collected through different surveillance systems.

The magnitude and intensity of the COVID-19 crisis led to increased possibilities in terms of data collection and analyses or linkage between databases. Existing routine surveillance systems were enhanced, with automation of data extraction, leading to an exhaustive, nearly real time national laboratory-based surveillance for cases (4). Novel surveillance systems were also developed, including registration of all hospitalizations for COVID-19 (5), a performant system for an accurate estimation of COVID-19 mortality in health care settings (including nursing homes) (6), surveillance of SARS-CoV-2 in waste water (7), genomic surveillance (8) and data collection on COVID-19 cases in schools and in nursing homes (9). Links with academic partners were reinforced, for instance for scenarios analysis and modeling (10). In addition, other sources of data, *a priori* not directly linked to public health, also provided information: mobile phone network provider or Google data informed on population mobility which was used to assess behavior and contacts among individuals (11); and passenger locator forms, which were mandatory in Europe over an extended period, allowed for monitoring travelers.

All data were collected independently from each other and following different flows. When possible, data were gradually integrated into the Healthdata.be platform, a system for standardizing the flow of health-related scientific data (12). All data, coming from the Heatlhdata.be platform or not, were collated by epidemiologists from Sciensano, analyzed and presented in a comprehensive weekly epidemiological report (13). These reports were publicly available to health authorities and to the general public, but because of the diverse sources of data, the interpretation of the epidemiological situation was complex. A simple way to communicate on the epidemiological situation and the risk for public health, as well as a link of a given risk level with specific control measures were requested by the authorities. For this purpose, different attempts were made to set up a dynamic management tool acting as a “COVID-19 barometer.” Starting from May 2020, different expert groups or authorities proposed different systems, that were not fully implemented in practice. From December 2020 onwards, it has been the responsibility of the RAG to coordinate the management tool.

Here, we describe the successive tools that were implemented and how they were used by the RAG in the COVID-19 context. We discuss lessons learned from these tools and what they can bring to future surveillance and policy making.

## Policy options and implications: use of management tools

### Successive management tools used in Belgium during the COVID-19 pandemic by the RAG, selection of indicators and thresholds

Once a week, the COVID-19 epidemiological situation was discussed with a core group of experts from the RAG. Between August 2020 and December 2022, a total of 122 evaluations were performed. The epidemiological evaluation was based on a wide range of indicators, which resulted from the enhanced surveillance efforts (Table 1).

**Table 1:** Indicators used for the weekly evaluation of the epidemiological situation.

Indicators	Period used	Data sources and references
Number of new COVID-19 cases and Rt of cases	August 2020–present	COVID-19 test database (4)
Number of COVID-19 positive tests and positivity rate	August 2020–present	COVID-19 test database (4)
Number of self-tests sold in pharmacies and positivity rate of self-tests	April 2021–July 2023	COVID-19 test database and APB (Belgian Association of Pharmacies)
Number of admissions in hospitals for COVID-19 and number of beds occupied (total and ICU)	August 2020–July 2023	COVID-19 hospital database (5)
Doubling time of number of hospitalizations for COVID-19	August 2020–July 2023	COVID-19 hospital database (5)
Number of deaths due to COVID-19	August 2020–July 2023	COVID-19 mortality database (6)
Circulating SARS-CoV-2 variants	December 2020–present	Molecular surveillance (8)
SARS-CoV2 viral load in waste water	September 2021–present	Wastewater surveillance (7)
Number of consultations for COVID-19 (suspicion) in GP practices	October 2020–present	Sentinel GP network and GP barometer (24, 25)
Number of cases, hospitalizations and death due to COVID-19 in nursing homes	October 2020–July 2023	Surveillance in nursing homes (9)
Number of children absent in schools	December 2020–May 2022	Surveillance in schools (26)
Number of arriving travelers, by country of departure	January 2021–February 2022	Passenger Locator Forms (PLF)
Place and source of infections	December 2020–November 2021	Contact tracing database (27)
Mobility of Belgian citizens	August 2020–March 2022	Mobile operator Proximus and Google databases
Vaccination coverage	March 2021–present, when relevant	LinkVacc database (28)
International situation	August 2020–present, when relevant	ECDC, WHO

From December 2020 onwards, the outcome of the epidemiological assessment was translated into a risk level. Three successive management tools were implemented by the RAG over time to define such risk levels (Table 2). These tools were mainly based on indicators reflecting viral circulation and pressure on health care (both first line and second line care). For each tool, indicators were chosen based on (i) their relevance depending on the phases of the epidemic and the objective of measures taken (reduction of the impact of infection at individual level and/or prevention of healthcare system overload), (ii) the testing strategy at a given time, (iii) the evolving population immunity and (iv) the evolving knowledge of the RAG members. For each indicator, thresholds were defined upon discussion with the group of experts, built on acquired experience as well as from quantitative evidence based on earlier waves and model-based relationship between earlier (e.g., infections) and later indicators (e.g., hospitalization and ICU admission). Additional indicators, such as the results of the waste water surveillance, the genomic surveillance, the (excess) mortality or the international situation, were not part of the tool *per se*, but were included in the global evaluation when relevant.

**Table 2:** Indicators and thresholds defined for the successive epidemic management tools.

			Indicators							**Communication**
Name	Date	Levels	14-day incidence cases	Positivity Rate	Rt	Consultations at GP practices	7-day incidence hospitalizations	ICU occupancy	Doubling time	
Epidemic management tool 1	Dec 2020 – July 2021	Control phase	<100/100000	<3%	<1					Weekly COVID-19 bulletin
		Lock-down phase plan A	100–300/100000	>3%			>4,5/100000			
		Lock-down phase plan B	>300/100000	increasing trend			>4,5/100000 and increasing trend			
		Lock-down phase plan C				GP capacity overloaded	>9/100000			
Epidemic management tool 2	July 2021 – Jan 2022	Level 1: Risk management	<20/100000	0–3%	<1,000	<25/100000	<2/100000	<15%	>100 d	RAG epidemiology report and weekly COVID-19 bulletin
		Level 2: Risk management	20–99/100000	0–3%	<1,000	25–49/100000	2–4,5/100000	15–24%	21–100 d	
		Level 3: crisis management	100–299/100000	3,1–6%	1,000-1,299	50–99/100000	4,6–6/100000	25–49%	16-20d	
		Level 4: crisis management	300–399/100000	6,1–10%	1,300-1,500	100–125/100000	6,1–9/100000	50–59%	5–15 d	
		Level 5: crisis management	>400/100000	>10%	>1,500	100–125/100000 + increasing trend	>9/100000	>60%	< 5d	
Epidemic management tool 3	Jan 2022 - present	Level 1	<200/100000		<1,000	<50/100000	<4/100 00	<15%		RAG epidemiology report and weekly COVID-19 bulletin
		Level 2	200–499/100000		1,000-1,299	50–99/100000	4–9,9/100000	15–24%		
		Level 3	≥500/100000		≥1,300	≥100/100000	≥10/100000	≥25%		

The first epidemic management tool, which was used by the RAG from December 2020 to July 2021, was initially proposed by the “*Corona Commissariat,”* a multidisciplinary coordination committee put in place in Belgium between October 2020 and April 2022 in order to, among others, coordinate communication between the different political authorities at the federal and federated levels, provide support to policy decisions and their implementation, and monitor the social and economic impact of the measures taken (14, 15). The tool consisted of two phases: a control phase and a lock-down phase. The thresholds to move from one phase to another were based on the 14-day incidence in cases, the positivity rate and the Rt calculated based on the number of cases, as these were the indicators relevant at the time. In the control phase, case management was done at a local level (analysis of clusters in collectivities, whereabouts, analysis of local increases of number of cases at municipality level up to the smallest administrative unit). The lock-down phase was reached when viral circulation exceeded the threshold of a national 14-day incidence of 100 new cases per 100,000 inhabitants. In that situation measures were expected to be applied in order to reduce viral circulation and return to the control phase. In addition, the lock-down phase was further subdivided into 3 plans (A, B and C) since, within this phase, the situation could stabilize/improve or on the contrary evolve unfavorably and require additional measures. The measures to be applied for each phase or plan were defined by a multidisciplinary group of experts (GEMS, Group of Experts for the Management Strategy for COVID-19, reporting to the Corona Commissariat), advising the Belgian authorities, complementary to the RAG.

The second epidemic management tool was in use from July 2021 to January 2022. This tool evolved from the first and consisted of five alarm levels, the first two constituting the “risk management phase” and the later three the “crisis management phase.” Similar to the control phase of the first epidemic management tool, the objective of the risk management phase was to limit, as much as possible, large-scale national measures and to contain localized outbreaks with appropriate measures taken at local level (administrative unit of municipality). The three levels of the crisis management phase were linked to the same measures as defined in plans A, B and C of the lock-down phase of the first epidemic management tool (3). The five levels of this tool were defined based on early indicators (14-day incidence of cases, positivity rate, Rt calculated based on the number of cases, number of consultations for suspicion of COVID-19 at General Practioner (GP) practices) as well as late indicators (7-day incidence of hospital admissions, occupancy of ICU beds and doubling time of hospital admissions). Compared to the first epidemic management tool, late indicators were included in the second version of the management tool because, as the epidemic evolved with increasing immunity in the population through vaccination or past infections, the impact of infections on individuals and society was reduced, and more importance was given to the severe infections with burden on hospitals.

The third epidemic management tool was in place from January 2022 to August 2023. This tool was developed upon request from the authorities who wished for a simplified management strategy that would indicate, on the basis of progressive, balanced and conditioned measures, how an epidemiological baseline situation could be achieved. The objective of this tool was also to provide clear communication toward the general public regarding public health measures. For this reason, the number of levels was reduced to three. Management level 1 was defined as an epidemiological situation under control, with virus circulation remaining at low level and without impact on the health care system. Management level 2 was reached when the viral circulation increased and pressure on the health care system was reported; measures were then needed to reverse the trend. Management level 3 reflected a situation of high virus circulation with an important risk of health care system overload (3). Because of the increasing use of self-tests instead of tests in laboratories, for which results were not systematically reported, the incidence in the number of new cases became less reliable (16). The three levels of this tool were therefore primarily defined based on hospital indicators (7-day incidence of hospital admissions and occupancy of ICU beds) as well as the number of consultations at GP practices for suspected COVID-19 (as an earlier indicator). Supporting indicators included the positivity rate for symptomatic patients, the Rt and the 14-day incidence of the number of cases.

**Figure 2:**
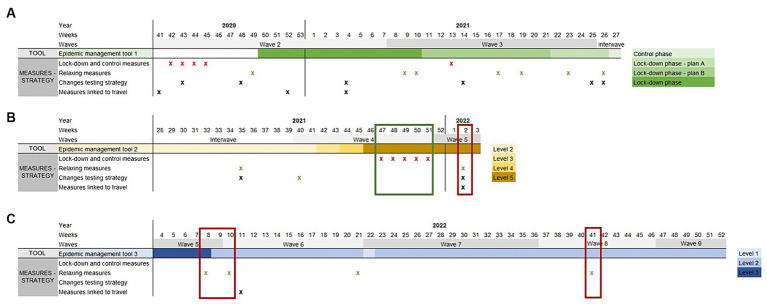
Parallel evolution of tools set up by the RAG for epidemic management and measures taken, between week week 41 of 2020 and week 52 of 2022. For each week, the level, as defined by the epidemic management tools, is indicated (panel **A**: Epidemic management tool 1: control phase and lock-down phase, later divided in 3 plans (plan A, B and C); Panel **B**: Epidemic management tool 2: levels 1 to 5; Panel **C**: Epidemic management tool 3: levels 1 to 3). The time when a measure was taken or a change in strategy applied is indicated by a cross (red cross: control measures; green cross: relaxing measures; black cross: changes in testing strategy or measures linked to travel). The red boxes provide examples of discrepancies between the management level decided by the RAG and the measures taken, the green box shows an example of management level and measures taken aligned.

As shown in Figure 2, since December 2020, a risk level was thus applied to the epidemiological situation on a weekly basis, based on the management tools described above. Figure 2 also indicates the measures that were taken or relaxed over time. A discrepancy between the management level proposed by the RAG and the measures taken by authorities was often, but not always, observed. For instance, in October–December 2021, when Belgium faced a wave of COVID-19 cases and hospitalizations linked to the Delta variant, the RAG recommendations and the authorities decisions were aligned and recommended measures were applied. On the other hand, in January 2022, all indicators were on the rise due to the Omicron variant and the epidemiological situation was evaluated by the RAG as “alarm level 5” (highest level). At the same time, at authorities’ level, it was decided to stop testing of asymptomatic high-risk contacts (to prioritize testing for symptomatic patients) and stop quarantine for fully vaccinated high-risk contacts. In February and March 2022, measures were progressively relaxed for events, restaurants, night clubs, etc. while the management level was, respectively, set by the RAG at level 3 in February (highest level) and at level 2 in March.

### Strengths and weaknesses of the Belgian system: regular evaluation combined to management tools

The establishment of an independent group of experts, within an existing structure for risk assessments (the RAG), allowed for a comprehensive interpretation of the epidemiological situation based on all available data and on a regular basis. In addition, within the management tools, the results of surveillance data were compiled in a clear manner and translated, weekly, into one risk level. The opinion of the experts participating to the RAG was important to reach a conclusion; in addition to the indicators, they took into account the expected future evolution of the epidemic and the link between the management level and the measures needed. Altogether, the system provided the authorities with scientific-based information translated into a risk-level, enabling them to take decisions which ultimately could control the evolution of the epidemic (reduction in cases, hospitalizations and deaths).

The strengths of this system were to (i) provide a simple and clear way of communication between experts and health authorities integrating various types and sources of data, and (ii) maintain a continuous and structured analysis, based primarily on objective indicators and thresholds. Although several management tools followed one another, the indicators and thresholds remained comparable, offering a stable framework for the evaluation and understanding of the epidemiological situation. This was exemplified when the Omicron variant replaced the Delta variant, the relationship between the indicators linked to cases and those linked to hospitalizations changed, highlighting the lower severity of the disease caused by the Omicron variant.

The system also showed some limitations. First, although one of the objectives of the management tools was to inform decision making, and despite the fact that in Belgium authorities heed the scientific evidence produced, a simple linkage of a risk level to a defined set of measures could in practice rarely be applied. This could be explained by the fact that the evaluation and the management tool focused on the epidemiological situation, while the decision process also had to take into account other factors such as the socio-economic situation and the mental health of the population (17). Second, the expectations from the authorities and the experts of the RAG regarding the management tool sometimes differed. Even though there was a clear will from authorities to base decision making on scientific evidence, they wished for a quantitative system resulting in a simple two level switch (on/off), whereas the RAG experts considered the situation as more complex, hence requiring a qualitative global interpretation in addition to the quantitative evaluation. Third, since the management tool was based on a series of indicators as well as a qualitative interpretation, and because several different management tools were successively set up, understanding the process was not always easy for the general population. Thus, the communication benefit offered by the management tool was of interest for the authorities but less so to the general population.

### Development of management tools in other European countries during the COVID-19 pandemic

In order to feed the discussion within the RAG in Belgium on the usefulness of a management tool during epidemics, a consultation of practices regarding the use of a tool for the management of the COVID-19 pandemic in other European countries was performed in May 2023, through the Population Health Information Research Infrastructure portal (PHIRI) (18). Eleven of the 14 EU countries who replied mentioned using or having used a management tool to monitor the COVID-19 epidemiological situation. All countries using a management tool based it on similar indicators as Belgium, namely the incidence of new cases, the incidence of new hospitalizations and the ICU occupancy. Ten countries mentioned associating a risk level to specific public health measures. However, it remains unclear how/if, in practice, these measures were implemented according to the defined risk level.

## Recommendations for further use of a management tool

Progressively, surveillance of COVID-19 in Belgium has been integrated into a broader monitoring, including influenza and other respiratory infections (19). The experience gathered during the COVID-19 crisis in terms of data management and data use for risk assessment founded the scheme for the current assessment of the epidemiological situation of these infections.

- It is important to invest in automated near real time data collection systems and performant data flows. Although an enhanced data collection, as done during COVID-19 pandemic, is not sustainable in the longer term, an easy reactivation when needed must be assured. In addition, some systems that were set up for COVID-19, such as automated data extraction from laboratory-based surveillance, should be continued and generalized to other pathogens in order to ensure timeliness, completeness and quality of data.
- Artificial intelligence approaches could be implemented to improve the analysis of large volumes or different data types (20, 21)
- Developing a management tool with risk levels can be considered to assess the severity of the epidemiological situation of respiratory infections and to inform public health preparedness and response.
- The risk levels should be defined by various indicators, combining different data sources, to gather early signals as well as to assess the severity of the situation.
- A set of measures and actions can be associated to each level, including public health mitigation measures and actions linked to surveillance intensity. Dialog between policy makers and surveillance / public health professionals is essential to ensure the applicability of such measures.
- Maintaining a stability of levels is important for clarity and endorsement by the general public.
- An evaluation of the management systems developed by each individual country during the COVID-19 pandemic should be carried out to define the most efficient system for risk assessment and risk management of epidemics.

These recommendations are in line with several initiatives put in place at international level, in the aftermath of the COVID-19 crisis, to support preparedness plans for pandemic and epidemic threats. The WHO has for instance developed an approach for the surveillance of epidemic threats called the WHO Hub for Pandemic and Epidemic Intelligence. It combines information from traditional surveillance, event-based surveillance, participatory or community surveillance, and on-the-ground investigations with contextual information, to generate an assessment of public health risk (21). WHO also issued a framework for resilient surveillance for respiratory viruses of epidemic and pandemic potential (“Crafting the mosaic”) where it is stressed that multiple approaches (different systems, investigations or studies) are needed together to provide essential information to policy makers (22). Taken together, these initiatives highlight the importance of collaboration between different instances (government institutions, non-governmental organizations, academia, private sector, civil society) and integration of the different surveillance systems (23).

## Conclusion

The important changes developed for the surveillance of COVID-19 serve current data collection and risk assessments for respiratory infections. In Belgium, enhanced data collection has not been maintained in a continuous way but could be reactivated if needed. An integrated surveillance for respiratory infections has been implemented, based on sentinel surveillance at the level of general practices (number of consultations, sentinel sampling) and hospitals (number of hospitalizations, severity of disease, sentinel sampling). Based on the COVID-19 experience, an adapted management tool for respiratory pathogens has been developed to facilitate risk assessment, communication toward authorities and propose recommendations for mitigation measures depending on a risk level in the current winter season (24). An evaluation of this tool is foreseen.

## Author contributions

GM: Conceptualization, Formal analysis, Writing – original draft, Supervision. VL: Conceptualization, Formal analysis, Writing – review & editing. GS: Conceptualization, Formal analysis, Writing – review & editing, Supervision. CB: Formal analysis, Writing – review & editing. CF: Methodology, Writing – review & editing. NH: Formal analysis, Writing – review & editing. PH: Methodology, Writing – review & editing. GM: Formal analysis, Methodology, Writing – review & editing. JaS: Formal analysis, Writing – review & editing. CK: Formal analysis, Writing – review & editing. SB: Formal analysis, Writing – review & editing. RB: Data curation, Writing – review & editing. JoS: Formal analysis, Writing – review & editing, Supervision TL: Conceptualization, Formal analysis, Writing – original draft, Supervision.
